# Intramedullary Nailing Versus Plate Fixation for the Treatment Displaced Midshaft Clavicular Fractures: A Systematic Review and Meta-Analysis

**DOI:** 10.1038/srep34912

**Published:** 2016-10-20

**Authors:** Nasir Hussain, Corey Sermer, Parker J. Prusick, Laura Banfield, Amit Atrey, Mohit Bhandari

**Affiliations:** 1 Central Michigan University College of Medicine, CMED Building 1280 S. East Campus St. Mt. Pleasant, MI 48859, USA; 2 Division of Orthopaedic Surgery, Mount Sinai Hospital, University of Toronto, 600 University Avenue Toronto, Ontario M5G 1X5, Canada; 3 Health Sciences Library, McMaster University 1280 Main St. W., HSC 2B Hamilton, Ontario L8S 4K1, Canada; 4 West Suffolk Hospital Bury St Edmunds, Suffolk IP33 2QZ, United Kingdom; 5 Division of Orthopaedic Surgery, Department of Surgery, McMaster University & Centre for Evidence-Based Orthopaedics 293 Wellington Street North, Suite 110 Hamilton, Ontario, L8L 8E7, Canada

## Abstract

The two commonly performed surgical techniques used to repair displaced midshaft clavicle fractures are plate fixation or intramedullary nailing; however, despite recent evidence, the optimal method to treat such fractures remains a continued topic of debate. A meta-analysis of randomized controlled trials (RCTs) was conducted to evaluate long term function, complications, and operative duration in adult patients receiving intramedullary nailing in comparison to plating. Seven RCTs and three quasi-randomized trials were included. No significant difference was found in long-term function between the two groups (MD: −0.66, 95% CI: −2.03 to 0.71, I^2^ = 62%, p = 0.34). Patients who received plating had a 2.19 times increased risk of treatment failure, but this failed to reach significance (95% CI: 0.93 to 5.15, I^2^ = 0%, p = 0.07). The risk of non-operative complications was 2.11 times higher in patients who received plating and this reached statistical significance (95% CI: 1.38 to 3.23, I^2^ = 53%, p = 0.0006). Finally, plating significantly prolonged operative duration by 20.16 minutes (95% CI: 16.87 to 23.44, I^2^ = 56%, p < 0.00001). Our results suggest that intramedullary nailing and plating provide equivalent long-term functional outcomes; however, plating may lead to a higher risk of treatment failure and non-operative complications.

Clavicle fractures account for approximately 2–4% of all adult fractures and about half of all fractures in the shoulder girdle^1^,^2^. The most common cause for a clavicle fracture is a direct blow to the shoulder or a fall on an outstretched arm^3^,^4^. The majority (69–82%) of these fractures occur in the middle third of the clavicle, or the midshaft^2^,^3^,^5^. Traditionally, these fractures were treated non-operatively as rates of nonunion were less than 1% after conservative treatment^5^,^6^. Recently, however there is increasing evidence that conservative treatment of displaced midshaft clavicle fractures results in higher rates of nonunion and deficits in shoulder muscle strength and endurance^7^,^8^. It has therefore been suggested that surgical intervention for these fractures should be considered due to lower rates of nonunion and greater patient satisfaction^9^,^10^,^11^,^12^.

There are two commonly performed surgical techniques used to repair displaced midshaft clavicle fractures: (1) open reduction and plate fixation and (2) intramedullary nailing^13^,^14^,^15^. Although both techniques have been proven to reduce the rates of nonunion and enhance functional outcomes for patients with displaced midshaft clavicle fractures^16^, they are both associated with a different set of drawbacks. Implant irritation or implant failure requiring revision has been consistently reported following open reduction and plate fixation of displaced midshaft clavicle fractures^17^. Additionally, this procedure has been associated with other minor complications such as superficial infections and plate loosening^18^. Although better cosmetic results have been reported after intramedullary fixation (due to a smaller incision), migration of the nail and implant irritation have been documented^19^,^20^.

The optimal method to treat displaced midshaft clavicle fractures remains a continued topic of debate. Despite the large number of individual studies published on the topic, it is still relatively unknown as to which surgical intervention provides better long-term functional outcomes and reduces overall complication rates. Recent reviews have been conducted in an attempt to determine which technique is superior, however they have either been inconclusive due to the limited number of published studies or have lacked adequate pooling due to insufficient study reporting^13^,^15^,^21^. Moreover, several recent trials have been conducted on this topic, thereby creating an opportunity to provide a more precise estimate of clinical outcomes. Thus, the overall objective of this systematic review and meta-analysis is to compare the long term functional outcomes and complication rates of plate fixation versus intramedullary nailing techniques for the treatment of displaced midshaft clavicle fractures in adult patients.

## Materials and Methods

### Criteria for Study Inclusion

Any randomized or quasi-randomized trial allocating adult patients (≥18 years old) undergoing repair of midshaft clavicle fractures to either plating or intramedullary nailing was considered for eligibility. In cases where there was no mention of randomization, the author of the study was contacted for more information. In regards to quasi randomization, any trial that made mention of a quasi-random method of allocation such as by date, case number or date alteration was considered. Studies published in languages other than English were excluded.

### Search Methods for Identification of Studies

A systematic search strategy was created by a librarian (L.B.) for various online databases including Cochrane Library, EMBASE, Medline, Evidence-Based Medicine (EBM) Reviews, and Database of Abstracts of Review Effects (DARE) from inception to January 1, 2016 for both published and unpublished articles. Two independent reviewers (N.H. and C.S.) first screened the search results from each of the databases by title and abstract alone. Potentially relevant articles were then retrieved and subsequently screened by way of full text eligibility. The bibliographies and citations of each relevant article were reviewed in order to ensure that no article was missed.

### Primary and Secondary Outcomes

The primary outcomes for this meta-analysis were overall long term function (≥12 months), complications requiring non-routine surgery, and overall adverse events *not* requiring non-routine surgery. The secondary outcome was to compare operative duration between plating and intramedullary nailing.

Complications requiring non-routine surgery were considered to be an indication of treatment failure. We defined treatment failure as complications such as non-union, malunion, hardware failure, re-fracture, or any other indication for revision surgery that was otherwise non-routine. We did not include routine pin or plate removal in this category unless there was indication for treatment failure. We used this definition due to the inconsistency in reporting between included studies.

Adverse events not requiring routine surgery were considered to be wound infection/dehiscence, cosmetic alterations such as skin deformity or skin alteration, asymptomatic non-union/malunion, hardware irritation/symptomatic hardware, or any other related minor complication. Pin removal was considered to be routine and not a complication if there was no indication provided by the study for removal. These complications were chosen since they were considered to be patient important and most commonly reported by the included studies.

### Assessment of Methodological Quality and Risk of Bias

The methodological quality for each included study in this meta-analysis was evaluated using the Cochrane Tool For Assessment Of Risk Of Bias^22^. Questions in this tool related to randomization, blinding, and outcome data reporting. Two reviewers (N.H. and C.S.) independently assessed the methodological quality of each included article. The authors of the included randomized controlled trials (RCTs) were also contacted to provide further information regarding the methodology if necessary. An un-weighted κ calculation was used to quantify the agreement between the two reviewers. κ values between 0.40 and 0.59 were considered to be fair agreement, values between 0.60 and 0.74 reflected good agreement and values greater than 0.75 represented excellent agreement^22^.

Recommendations made by the Grade Working Group were utilized to investigate the overall quality of evidence for each pooled outcome^23^. RCTs were initially graded as high quality evidence and were downgraded accordingly based upon the overall risk of bias, inconsistency between studies, indirectness of evidence, imprecision, and publication bias.

### Measurement of Treatment Effect

Shoulder function is a continuous outcome that can be measured through a wide variety of well-validated questionnaires including the Constant-Murley Shoulder Outcome questionnaire and, Disabilities of the Arm, Shoulder and Hand questionnaire (DASH), and the American Shoulder and Elbow Surgeons Standardized Shoulder Assessment Form (ASES). The Constant-Murley score is one of the most commonly used shoulder function questionnaires and has been thought to be more specific for shoulder function than the DASH score^15^. As such, to enhance interpretability of the results, all scores of the functional questionnaires were first rescaled to natural units of the Constant-Murley score using the methods described by Thorlund *et al*.^24^. Following this, a mean difference (MD) and 95% Confidence Interval (CI) were calculated for this outcome. An MD and 95% CI was also calculated for operative duration, which is measured in the unit of minutes.

Complications requiring non-routine surgery and adverse events not requiring surgery are dichotomous outcomes. As such, a risk ratio (RR) with 95% CI was calculated for this outcome. Data for this outcome only included patients that were available at final follow-up and not those that were initially randomized.

### Assessment of Heterogeneity and Sensitivity Analysis

Heterogeneity was calculated using an I^2^ statistic test, with the threshold for conducting subgroup analysis for this data being an I^2^ > 40%. Per the Cochrane Handbook for Systematic Reviews, an I^2^ greater than 40% suggests that heterogeneity may be present^22^. Additionally, subgroup analysis was only conducted when greater than two studies were present in a specific subgroup. If this was the case, heterogeneity was explored on the basis of overall study design (quasi-randomized vs. randomized trial).

Sensitivity analysis was performed by sequentially excluding the data from quasi-randomized trials sequentially, one at a time. Quasi-randomized trials have an inherently high risk of bias, thereby potentially influencing the treatment effect.

### Data Synthesis

When data could be appropriately pooled, a meta-analysis was performed using the Mantel-Haenszel Random-Effects Model since there was expected heterogeneity between the included studies. P-values less than 0.05 were considered to be significant. For continuous outcomes, an MD value less than 0 represented an overall score change favoring intramedullary nailing. Conversely, an MD value greater than 0 represented a overall score change favoring plating.

## Results

### Study Inclusion

**Figure 1:**
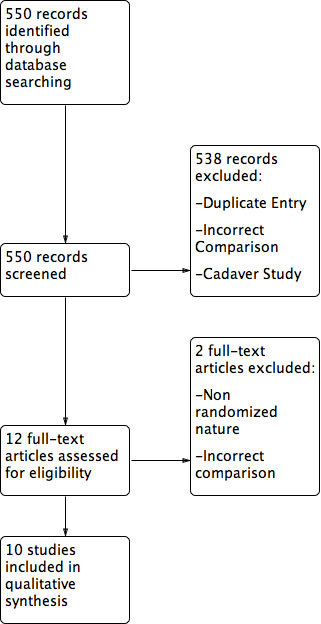
Study inclusion flow diagram.

A systematic search strategy was created and a total of 550 articles were assessed based on title and abstract for eligibility. The full search strategy can be viewed in Appendix A. After initial title and abstract review, 12 articles were assessed for full text eligibility. Two of these articles were excluded due to their non-randomized nature or lack of intervention/comparator of interest. Thus, a total of seven randomized^12^,^25^,^26^,^27^,^28^,^29^,^30^ and three quasi-randomized trials^31^,^32^,^33^ were included in this review. The raw agreement between the independent reviewers for full text eligibility was found to be 90.5% and the un-weighted κ was 0.85, which represents excellent agreement. The full flow diagram of study inclusion can be viewed in Fig. 1.

### Study Characteristics

A total of seven randomized^12^,^25^,^26^,^27^,^28^,^29^,^30^ and three quasi-randomized trials trials^31^,^32^,^33^, which assessed displaced midshaft clavicle fractures, were included in this review. The full characteristics of the included studies can be viewed in Table 1. All studies were conducted between the years 2007 and 2015 and were conducted outside North America. All of the studies reported a greater than 50% proportion of males in the study population. The mean age of the patients ranged from 29.3 to 58 years of age. Although all studies used plate fixation as comparison, the nailing methods varied. Specifically, five studies utilized a titanium elastic nail (TEN)^25^,^28^,^29^,^32^,^34^, two studies utilized a Knowles pin^27^,^31^, one study utilized a Rockwood pin^26^, one study utilized a collarbone pin^30^, and one study did not specify the type of intramedullary nail used^33^.

### Risk of Bias Assessment

**Figure 2:**
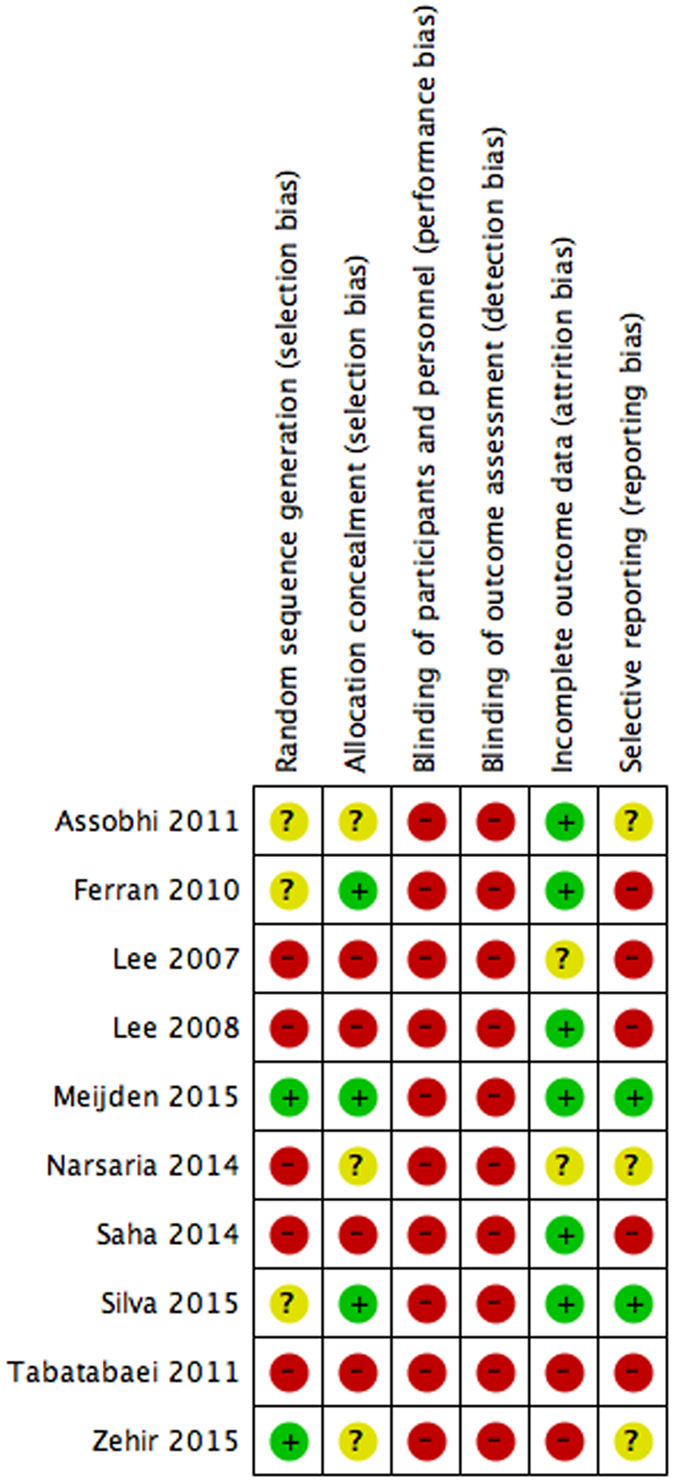
Study Characteristics.

The risk of bias assessment of all included studies can be viewed in Fig. 2. All studies were classified as having an unclear to high risk of bias across the majority of parameters assessed. All studies were classified as high risk of bias for blinding of participants, study personnel or outcome assessors. Studies either did not describe whether blinding methods were used or explicitly stated that individuals involved with the study were not blinded. Several studies reported using concealed envelopes as a method of randomization; however, no mention was made in regards to opacity of the envelopes themselves. Two studies had a high percentage of incomplete data or loss to follow-up but did not describe whether the results varied in these missing patients^30^,^33^. Only two of the studies were pre-registered with clinical trial registries^28^,^29^. The overall un-weighted κ between the two independent reviewers was found to be 0.76. This represents an excellent level of agreement between the two reviewers.

### Long Term Function

**Figure 3:**
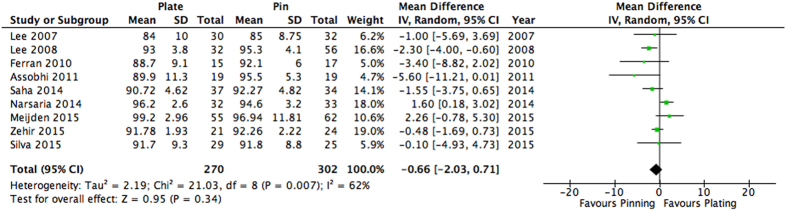
Pooled estimates with mean difference and 95% Confidence interval for long-term function (≥12 months) as assessed through the Constant-Murley score.

Overall shoulder function at one-year follow-up was assessed by ten studies^12^,^25^,^26^,^27^,^28^,^29^,^30^,^31^,^32^,^33^. One study only reported mean values without a measure of error or standard deviation, thus their results were excluded from the analysis^33^. Thus, a total of nine studies (n = 572) were included in this analysis. Eight studies reported shoulder function with the Constant Score^25^,^26^,^27^,^28^,^29^,^31^,^32^,^34^. One study^30^ solely used the DASH questionnaire and was rescaled to the Constant Score using the statistical methods described by Thorlund *et al*.^2^. Overall, it was found that long-term shoulder function at one-year follow-up did not significantly differ between patients receiving plating versus intramedullary nailing (MD: −0.66, 95% CI: −2.03 to 0.71, I^2^ = 62%, p = 0.34) (Fig. 3).

**Figure 4:**
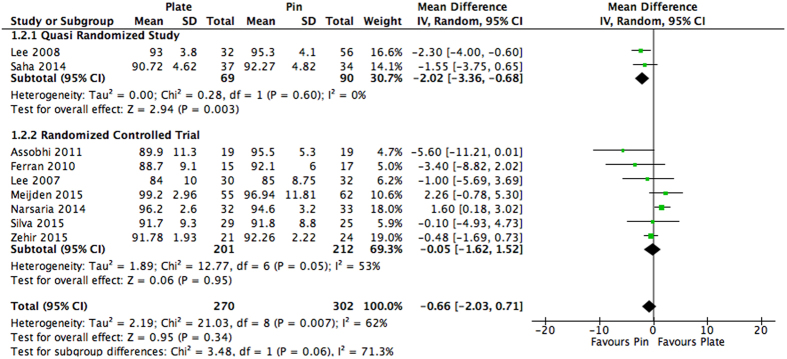
Subgroup analysis for long-term function (≥12 months) as assessed through the Constant-Murley score. Studies are categorized by study design. Values represented as a mean difference with 95% Confidence interval.

Heterogeneity was above our pre-defined cut off and thus subgroup analysis was conducted. Subgroup analysis was conducted based on study design. Quasi-randomized studies^31^,^32^ found a significant functional benefit when intramedullary nailing was used in comparison to plating (MD: −2.02, 95% CI: −3.36 to −0.68, I^2^ = 0%, p = 0.003). On the other hand, RCTs^25^,^26^,^27^,^28^,^29^,^34^ continued to show no significant difference between the two groups (MD: −0.05, 95% CI: −1.62 to 1.52, I^2^ = 53%, p = 0.95). The subgroups were not found to be significantly different (p = 0.06) (Fig. 4).

### Treatment Failure: Complications Requiring Non-Routine Surgical Intervention

**Figure 5:**
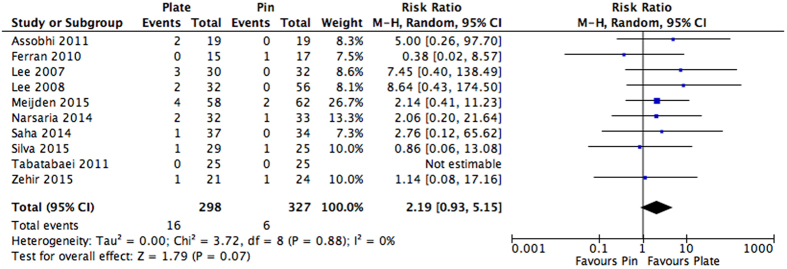
Pooled estimates presented as a risk ratio and 95% Confidence interval for overall complications requiring non-routine surgery for intramedullary nailing versus plating.

Several reasons were cited by the studies for non-routine surgical intervention, which included: re-fracture, symptomatic non-union/malunion, hardware failure, or mechanical failure. A full list of the complications requiring secondary operation can be viewed in Table 2. Patients receiving plate fixation were found to have a 2.19 times greater risk of developing complications requiring non-routine surgical intervention in comparison to those receiving intramedullary nailing; however, this result was not found to be statistically significant (RR: 2.19, 95% CI: 0.93 to 5.15, I^2^ = 0%, p = 0.07) (Fig. 5). These findings were robust to sensitivity analysis when the data from quasi-randomized studies was excluded (RR: 1.88, 95% CI: 0.74 to 4.77, I^2^ = 0%, p = 0.19).

### Adverse Events Not Requiring Surgery

**Figure 6:**
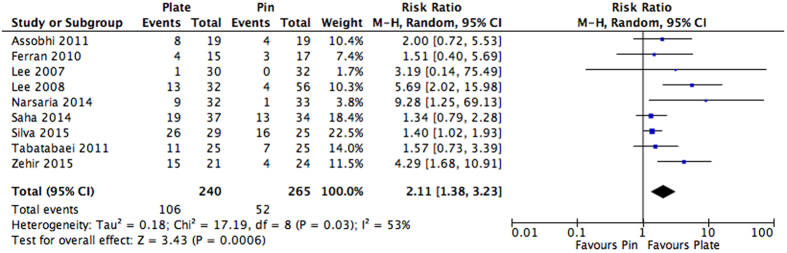
Pooled estimates presented as a risk ratio and 95% Confidence interval for adverse events not requiring surgery for intramedullary nailing versus plating.

Across the studies, there were several cosmetic and non-cosmetic adverse events that were reported. The most commonly reported adverse events included infection, hardware irritation, and inadequate cosmetic results; the full list of complications that did not require surgery can be found in Table 3. Of the ten studies that reported adverse events, nine could be pooled^25^,^26^,^27^,^29^,^30^,^31^,^32^,^33^,^34^. One study^28^ reported multiple adverse events per patient and did not provide sufficient patient level data. To avoid potential double counting of patients, we did not include its results into the overall analysis. It was found that patients receiving plate fixation had a 2.11 times greater risk of adverse events not requiring surgery in comparison to those receiving intramedullary nailing. This was found to be statistically significant (RR: 2.11, 95% CI: 1.38 to 3.23, I^2^ = 53%, p = 0.0006) (Fig. 6).

**Figure 7:**
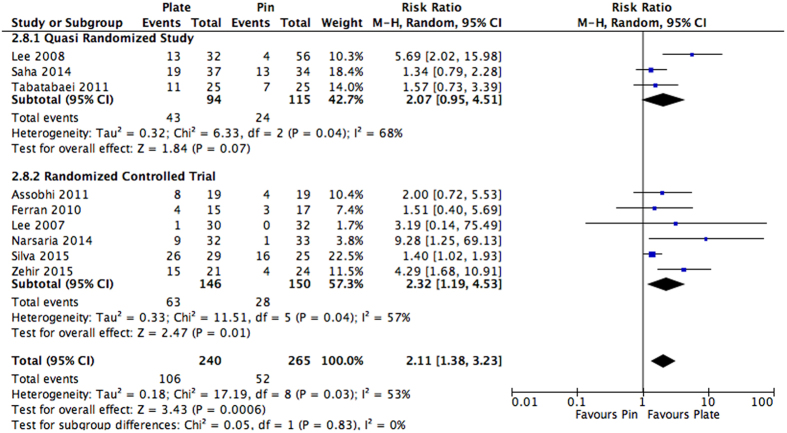
Subgroup analysis for adverse events not requiring surgery for intramedullary nailing versus plating. Studies are categorized by study design. Mean estimates are reported as a risk ratio and 95% Confidence interval.

Subgroup analysis was performed on the basis of study design. Quasi-randomized studies^31^,^32^,^33^ found a 2.07 times increased risk of adverse events not requiring surgery when plate fixation was used in comparison to intramedullary nailing; however, this was not found to be significant (RR: 2.07, 95% CI: 0.95 to 4.51, I^2^ = 68%, p = 0.07). In comparison, RCTs^25^,^26^,^27^,^29^,^30^,^34^ found a significantly greater risk of adverse events at 2.32 times when plate fixation was used in comparison to intramedullary nailing (RR: 2.32, 95% CI: 1.19 to 4.53, I^2^ = 57%, p = 0.01). The subgroups were not found to be significantly different (p = 0.83) (Fig. 7).

**Figure 8:**
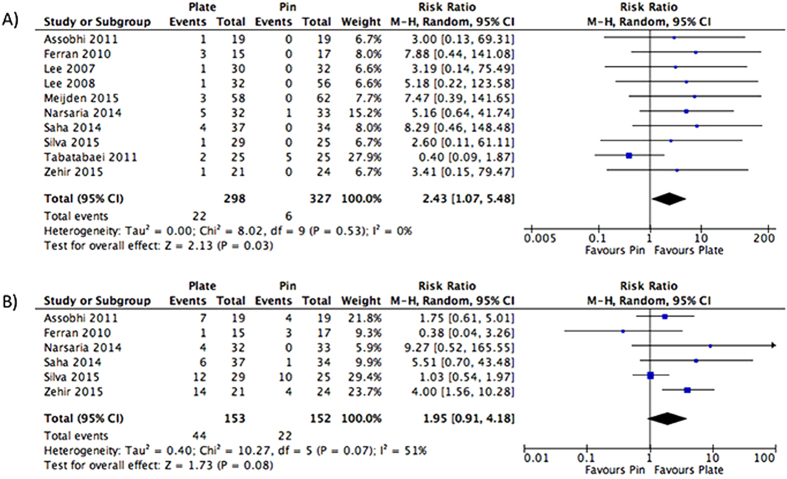
Pooled estimates represented as a risk ratio and 95% Confidence interval for rate of infection (**A**) and cosmetic dissatisfaction (**B**) in patients receiving intramedullary nailing in comparison to plating.

We further classified the adverse events not requiring surgery by specific type. Infection was reported by all ten studies. Patients receiving plate fixation had a 2.43 times greater risk of infection in comparison to intramedullary nailing, and this was found to be significant (RR: 2.43, 95% CI: 1.07 to 5.48, I^2^ = 0%, p = 0.03) (Fig. 8A). Commonly reported cosmetic dissatisfactions included hypertrophic scars, skin irritation, scar numbness, implant related pain and implant protrusion. Patients receiving plate fixation had a 1.95 times greater risk of cosmetic inadequacies in comparison to those receiving intramedullary nailing, but this failed to reach significance (RR: 1.95, 95% CI: 0.91 to 4.18, I^2^ = 51%, p = 0.08) (Fig. 8B). Other specific adverse events were not pooled due to differing definitions and lack of consistent reporting between the included studies.

### Operative Duration

**Figure 9:**
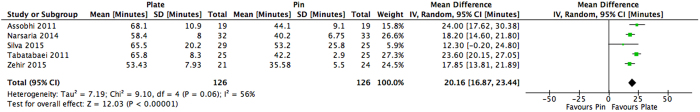
Operative duration represented as a mean in minutes with 95% Confidence interval in patient receiving intramedullary nailing versus plating.

Operative duration was assessed by a total of six studies^25^,^27^,^29^,^30^,^33^,^34^; however, five studies^25^,^29^,^30^,^33^,^34^ had sufficient information to allow for pooling. One of these studies^29^ reported only mean values without any measure of error or standard deviation. Upon contacting the authors, the SD was provided^29^. Plate fixation was found to significantly prolong the duration of surgery by 20.16 minutes in comparison to those receiving intramedullary nailing (MD: 20.16, 95% CI: 16.87 to 23.44, I^2^ = 56%, p < 0.00001) (Fig. 9). These findings were robust to sensitivity analysis when the data from the quasi-randomized study [34] was excluded (MD 18.80, 95% CI: 15.84 to 21.76, I^2^ = 23%, p < 0.00001).

## Discussion

The results of our meta-analysis suggest that intramedullary nailing and plate fixation provide similar long term functional outcomes and rates of treatment failure; however, plate fixation can lead to significantly greater risks of adverse events not requiring surgery such as infection. Expectedly, patients receiving plate fixation have longer operative times in comparison to those who receive intramedullary nailing. Sensitivity analysis did not alter the results and subgroup analysis did not reveal significant differences between quasi-randomized studies and RCTs. These findings may be limited by the smaller sample sizes and high level of methodological bias present across all studies. Although this may limit the external validity of our results, we believe that this is the best evidence to date on this popular topic.

As per the GRADE criteria and recommendations, there was a lack of high quality evidence across all evaluated outcomes (Appendix B). Evidence was downgraded due to several reasons, including the high level of risk of bias, imprecision and inconsistency of the results. Even though subgroup analysis was conducted; there was still considerable heterogeneity that was present across several outcomes.

Unlike previous systemic reviews and meta-analyses conducted on the topic, our review was able to sufficiently pool data across a wide range of outcomes and shed greater insights into clinically important outcomes. Our results also differ from previous reviews due to the additional studies included and larger amounts of pooling. Wang *et al*.^21^ were unable to categorize and create broad outcomes for complications and thus were only able to provide pooled estimates on specific adverse events. Inconsistent with our review, they also found no significant difference in regards to the risk of infection in patients with plate fixation in comparison to intramedullary nailing (RR: 1.09, 95% CI: 0.39 to 4.07, I^2^ = 0%, p = 0.71)^21^. Similarly, a Cochrane review conducted by Lenza *et al*.^15^ was limited since only two RCTs and two quasi-randomized trials were included that compared the interventions in question. We were able to include six additional randomized and quasi-randomized trials. In contrast to our review, they found a significant long term functional benefit when intramedullary nailing was used in comparison to plate fixation (MD: 4.46, 95% CI: 0.56 to 8.36, I^2^ = 0%, p = 0.025)^15^. Additionally, unlike our review they found no significant difference in adverse events not requiring surgery between the two groups (RR: 0.64, 95% CI: 0.39 to 1.03, I^2^ = 0%, p = 0.064)^15^. The differences in these results are likely attributed, again, to the larger number of studies included in our review. Furthermore, as our results suggest, a higher rate of adverse events not requiring surgery may be expected with plate fixation due to the relative invasiveness of the procedure. Zhu *et al*.^35^ also published a review with limited pooling and only five RCTs included. They found a functional benefit favoring intramedullary nailing; however, with a far greater effect size, we were able to suggest that this may be inaccurate. Finally, reviews by Duan *et al*.^13^ and Barlow *et al*.^36^ had a very small number of RCTs included and had very limited pooling.

The vast majority of clavicle fractures occur in the midshaft region with overall rates being reported as high as 82%^5^. Although non-operative management remains a viable option for many of these fractures, internal fixation is becoming increasingly common. The indications for fixation have expanded in response to evidence that non-unions occur as often as 15% in completely displaced mid-shaft clavicle fractures, and poor functional outcomes occur with fractures with greater than 20 mm of shortening or medialization^7^,^8^. Specifically, plating of such fractures remains popular amongst orthopaedic surgeons. The benefits of plate fixation have been widely reported^37^,^38^. For the physician, plating has been shown to be a reliable and relatively easy technique to learn and perform with the emergence of improved implants and better soft-tissue handling^9^. Post-operatively, plating also provides immense stability and strength, potentially enabling earlier rehabilitation^37^. Comparatively, intramedullary fixation of displaced midshaft clavicle fractures has also yielded good functional results and patient satisfaction^12^,^39^. Because of the minimally invasive nature of this technique, good cosmetic results have also been indicated, as a smaller incision is required^12^,^39^. Whether one technique is superior in comparison to the other has yet to be adequately addressed. Although our results suggest the potential comparable results achieved with each method, several important considerations need to be made such as cost, length of procedure, and ease of approach. Even though intramedullary nail fixation was found to provide a significantly lower rate of non-operative complications and a shorter operative duration, we still cannot provide a definitive recommendation as to which technique is optimal due to the lack of differences in functional outcomes. As such, we believe that both techniques are excellent options to treat displaced midshaft clavicle fractures, and thus the determination of which method to use should be left to the physician’s level of comfort with each approach.

### Strengths and Limitations

Overall, our review comes with several strengths and limitations. We were able to successfully pool data across all primary outcomes. Furthermore, due to the increased number of studies included, we were able to provide more precise estimates of effect. By creating broad outcomes, we also were able to provide more clinically relevant information in regards to overall complication rate and treatment failure. With this in hand, our review also comes with important limitations. Across all studies, there was a high level of methodological bias which could affect external validity. For instance, through subgroup analysis we found that long term function was significantly better with intramedullary nailing when only quasi-randomized trials were pooled; however, this difference was not observed when only RCTs were pooled. This may have been due to the inherent higher risk of methodological bias with quasi-randomized trials in comparison to RCTs. Beyond any methodological bias, there were inconsistencies in the type of intramedullary pin/nail used. The use of different materials, or type of pin, could have differing effects on functional outcomes and adverse effects, which has the potential to skew our data. Further investigation is required to determine if different pins lead to varying results. In addition, we were unable to account for the comminuted fractures, which may have been present. These types of fractures have been found to be best repaired using plate fixation, and thus their presence, and subsequent treatment with intramedullary nailing, may have skewed the results. Finally, the definitions used for certain complications varied greatly across each included study. This may have contributed to the relatively high level of unresolved heterogeneity in this review.

## Conclusions

This meta-analysis suggests that intramedullary nailing provides similar functional outcomes and rates of treatment failure in comparison to plate fixation; however, complications not requiring surgery appear to occur at a greater frequency when plating is used. Specifically, the rate of infection was found to be significantly greater when plate fixation was used. While choosing the most optimal method to treat midshaft clavicle fractures, clinicians should aim to maximize long term function while minimizing rates of revision and complications. Although plating appears to be the standard of care amongst orthopaedic surgeons, we believe that intramedullary nailing can provide a viable alternative that may be more cost effective due to significantly reduced operative times.

## Additional Information

**How to cite this article**: Hussain, N. *et al*. Intramedullary Nailing Versus Plate Fixation for the Treatment Displaced Midshaft Clavicular Fractures: A Systematic Review and Meta-Analysis. *Sci. Rep.* **6**, 34912; doi: 10.1038/srep34912 (2016).

## Supplementary Material

Supplementary Information

## Figures and Tables

**Table 1:** Study Characteristics.

Study	Country	Sample Size	% Males	Mean Age (Years)	Length of Follow-up	Intervention	Comparison	Primary Outcomes Assessed	Secondary Outcomes Assessed
Lee *et al*.^27^	Taiwan	62	58	59	30 m	Knowles Pin	Plate	Constant-Murley score	Operative time, wound size, incision length, hospital stay, analgesia use, complications, visual analog pain score
Lee *et al*.^31^	Taiwan	88	64.7	39.4	1 m, 2 m, 3 m, 4 m, 6 m, 12 m	Knowles Pin	Plate	Constant-Murley score	Shoulder score, incision length, operative time, analgesia use, complication rate, visual analog pain score
Assobhi *et al*.^25^	Egypt	38	86.8	31.45	6 w, 3 m, 6 m 12 m	Retrograde Titanium Elastic Nail	Plate	Constant-Murley score	Mean surgery time, blood loss, wound size, hospital stay, complication rates
Ferran *et al*.^26^	UK	32	84.3	29.3	2 w, 6 w, 3 m, 6 m, 12 m	Rockwood Pin	Plate	Constant-Murley score	Oxford shoulder score, union rate, complication rates
Tabatabaei *et al*.^24^	Iran	50	84	28.0	NR	Intramedullary Nailing	Plate	DASH score	Union time, Oxford shoulder score, complications
Narsaria *et al*.^34^	India	65	75.7	39.5	1 m, 2 m, 4 m, 6 m, 12 m, 18 m, 24 m	Titanium Elastic Nail	Plate	Constant-Murley score	Length of incision, operation time, blood loss, duration of hospital stay
Saha *et al*.^32^	India	71	84.5	33.1	2 w, 6 w, 3 m, 6 m, 12 m, 18 m, 24 m	Titanium Elastic Nail	Plate	Constant-Murley score	Operative time, intraoperative blood loss, wound size, cosmetic results, complications
Meijden *et al*.^28^	Netherlands	120	94.1	39	2 w, 6 w, 3 m, 6 m, 12 m	Titanium Elastic Nail	Plate	DASH score	Constant-Murley score, short form-36 questionnaires, likert scale for satisfaction with cosmetic result
Silva *et al*.^29^	Brazil	54	87	29.7	6 m, 12 m	Titanium Elastic Nail	Plate	DASH score	Constant-Murley score, time to fracture union, residual shortening, level of postoperative pain, percentage of satisfied patients, complication rates
Zehir *et al*.^30^	Turkey	45	57.7	32.7	1 m, then every 3 m thereafter	Intramedullary Pin	Plate	DASH score	Mean time of operation and flouroscopy, time of hospital stay, complications, radiographic bony union time

**Table 2:** Reported Complications requiring non-routine surgery (treatment failure).

Study	Intramedullary Nailing		Plating Fixation	
	Complication	Number of Patients (n = 327)	Complication	Number of Patients (n = 298)
Assobhi *et al*.^25^			Nonunion	1
			Refracture after implant removal	1
Ferran *et al*.^26^	Revision due to metalwork loosening	1		
Lee *et al*.^27^			Implant failure	2
			Nonunion	1
Lee *et al*.^31^			Implant failure	1
			Nonunion	1
Narsaria *et al*.^34^	Implant failure	1	Major revision surgery	2
Saha *et al*.^32^			Nonunion	1
Tabatabaei *et al*.^33^*				
Zehir *et al*.^30^	Implant failure	1	Implant failure	1
Silva *et al*.^29^	Nonunion	1	Implant failure	1
Meijden *et al*.^28^*	Implant failure	2	Refracture after implant removal	2
			Nonunion	1
			Implant breakage	1
**Total Patients**		6		16

*No major adverse events reported that required non-routine surgery.

**Table 3:** Reported adverse events not requiring surgery.

Study	Intramedullary Nailing		Plating Fixation	
	Complication	Number of Patients (n = 265)	Complication	Number of Patients (n = 240)
Assobhi *et al*.^25^	Permanent implant under skin	3	Hypertrophic scar	4
	Hypertrophic callus	1	Prominent implant under skin	3
			Infection	1
Ferran *et al*.^26^	Scar numbness	2	Infection	3
	Soft tissue irritation	1	Scar numbness	1
Lee *et al*.^27^			Infection	1
Lee *et al*.^31^	Symptomatic hardware	4	Symptomatic hardware	12
			Infection	1
Narsaria *et al*.^34^	Infection	1	Hypertrophic Scar	4
			Wound dehiscence	3
			Infection	2
Saha *et al*.^32^	Symptomatic hardware	12	Symptomatic hardware	9
	Hypertrophic callus	1	Ugly Scar	6
			Infection	4
Tabatabaei *et al*.^33^	Infection	5	Skin breakdown/Symptomatic hardware	8
	Asymptomatic nonunion	2	Infection	2
			Asymptomatic nonunion	1
			Asymptomatic non-union	1
Zehir *et al*.^30^	Cosmetic dissatisfaction	4	Cosmetic dissatisfaction	9
			Skin irritation	3
			Dysesthesia	2
			Infection	1
Silva *et al*.^29^	Implant-related pain	10	Implant bending	11
	Partial implant migration	5	Paresthesia	8
	Implant bending	1	Implant-related pain	4
			Partial implant migration	2
			Infection	1
Meijden *et al*.^28^*	Hematoma	6	Infection	3
	Transient neurapraxia	1	Hematoma	5
	Irritation due to implant protrusion	44	Irritation due to implant protrusion	25
	Implant failure	2	Implant breakage	1
			Nonunion	1
			Refracture after implant removal	2
**Total Patients**		52		107

*Study double counted patients. As such, since unique patient level data was not available, it is not included in the total patients estimate.
