# Supplementary material for: Intramedullary Nailing Versus Plate Fixation for the Treatment Displaced Midshaft Clavicular Fractures: A Systematic Review and Meta-Analysis
Source: Sci Rep. 2016 Oct 20;6:34912. doi: 10.1038/srep34912 (PMC5071841; doi:10.1038/srep34912)
Supplement: Supplementary Information [file srep34912-s1.doc]

**INTRAMEDULLARY NAILING VERSUS PLATE FIXATION FOR THE TREATMENT DISPLACED MIDSHAFT CLAVICULAR FRACTURES: AN UPDATED SYSTEMATIC REVIEW AND META-ANALYSIS**

Nasir Hussain MSc MD (Cand)^1^, Corey Sermer MSc (Cand)^2^, Parker J. Prusick MD (Cand)^1^, Laura Banfield MLIS^3^, Amit Atrey MBBS MRCS MSc FRCS^4^, Mohit Bhandari MD PhD FRCSC^5^

^1^Central Michigan University College of Medicine, CMED Building

1280 S. East Campus St.

Mt. Pleasant, USA

MI 48859

^2^ Division of Orthopaedic Surgery, Mount Sinai Hospital, University of Toronto,

600 University Avenue

Toronto, Ontario Canada

M5G 1X5

^3^ Health Sciences Library, McMaster University

1280 Main St. W., HSC 2B

Hamilton, Ontario, CANADA

L8S 4K1

**^4^**West Suffolk Hospital

Bury St Edmunds, Suffolk

United Kingdom

IP33 2QZ

^5^Division of Orthopaedic Surgery, Department of Surgery, McMaster University & Centre for Evidence-Based Orthopaedics

293 Wellington Street North, Suite 110

Hamilton, Ontario, Canada

L8L 8E7

**Appendix A:** Search Strategies

*Medline*:

1. Clavicle/ (4743)
2. collar bone*.ti,ab,kf. (26)
3. collarbone.ti,ab,kf. (27)
4. clavic*.ti,ab,kf. (8004)
5. or/1-4 (9618)
6. exp Fracture Fixation/ (49583)
7. Fracture Healing/ (10095)
8. fracture*.ti,ab,kf. (193654)
9. Bone Plates/ (13738)
10. Bone Nails/ (9400)
11. (bone adj2 (nail* or plat* or pin*)).ti,ab,kf. (2986)
12. (bone adj6 (nail* or plat* or pin*)).ti,ab,kf. (9491)
13. ((bone or intramedul*) adj3 (nail* or plat* or pin*)).ti,ab,kf. (10018)
14. Fractures, Bone/ (49656)
15. 6 or 7 or 8 or 9 or 10 or 13 or 14 (226327)
16. 15 and 5 (2854)
17. remove duplicates from 16 (2809)
18. random*.mp. (1022169)
19. rct*.mp. (24898)
20. randomized controlled trial.pt. (418424)
21. clinical trial.pt. (509363)
22. clinical trials as topic/ (180293)
23. controlled clinical trial.pt. (92318)
24. experimental trial*.mp. (1988)
25. clinical trial*.mp. (859192)
26. exp randomized controlled trial/ (418484)
27. exp clinical trial/ (860053)
28. ((singl* or doubl* or tripl* or trebl*) adj3 (blind* or mask*)).mp. [mp=title,
29. single-blind method/ (21725)
30. random allocation/ (87193)
31. double-blind method/ (136478)
32. or/18-31 (1671187)
33. 16 and 32 (197)

*EMBASE*:

1. clavicle/ (5204)
2. collar bone*.ti,ab,kw. (47)
3. collarbone*.ti,ab,kw. (59)
4. clavic*.ti,ab,kw. (9836)
5. or/1-4 (11432)
6. fracture/ (71460)
7. exp fracture fixation/ (72059)
8. fracture healing/ (19667)
9. fracture*.ti,ab,kw. (233367)
10. bone plate/ (12489)
11. bone nail/ (4059)
12. ((bone or intramedul*) adj36 (nail* or plat* or pin*)).ti,ab,kw. (39051)
13. or/6-12 (306757)
14. 5 and 13 (3365)
15. remove duplicates from 14 (3314)
16. random*.mp. (1209345)
17. rct*.mp. (37726)
18. controlled clinical trial/ (393750)
19. randomized controlled trial/ (392924)
20. clinical trial/ (858424)
21. experimental trial*.mp. (2308)
22. exp randomization/ (69096)
23. ((singl* or doubl* or tripl* or trebl*) adj3 (blind* or mask*)).mp. [mp=title, abstract, heading word, drug trade name, original title, device manufacturer, drug manufacturer, device trade name, keyword] (233631)
24. or/16-23 (1788776)
25. 24 and 15 (216)

*EBM Reviews*

1. clavic*.mp. [mp=ti, ot, ab, sh, hw, kw, tx, ct] (266)
2. collar bone*.mp. (4)
3. collarbone*.mp. (9)
4. or/1-3 (272)
5. fracture*.mp. (11434)
6. ((bone or intramedul*) adj3 (nail* or pin* or plat*)).mp. (1378)
7. 5 or 6 (11832)
8. 4 and 7 (140)
9. remove duplicates from 8 (137)

**Appendix B:** GRADE Tables for outcome quality

**GRADE FOR SHOULDER FUNCTION**

| **Quality assessment** | | | | | | | **№ of patients** | | **Effect** | | **Quality** | **Importance** |
| --- | --- | --- | --- | --- | --- | --- | --- | --- | --- | --- | --- | --- |
| **№ of studies** | **Study design** | **Risk of bias** | **Inconsistency** | **Indirectness** | **Imprecision** | **Other considerations** | **Plate Fixation** | **Intramedullary Nailing** | **Relative (95% CI)** | **Absolute (95% CI)** |
| Shoulder Function | | | | | | | | | | | | |
| 9 | randomised trials | serious ^1,2,3,4^ | serious ^5^ | not serious | serious ^6^ | none | 270 | 302 | - | MD **0.66 Points fewer** (2.03 fewer to 0.71 more) | ⨁◯◯◯ VERY LOW | CRITICAL |

**CI:** Confidence interval; **MD:** Mean difference

1. Lack of blinding across several parameters
2. Lack of adequate randomization and concealment techniques
3. Potential selective outcome reporting
4. High risk quasi-randomized studies included
5. High level of between study heterogeneity
6. Wide confidence interval

**ALL COMPLICATIONS REQURING SURGERY (TREATMENT FAILURE) AND GENERAL ADVERSE EVENTS (COMPLICATIONS NOT REQUIRING SURGERY)**

| **Quality assessment** | | | | | | | **№ of patients** | | **Effect** | | **Quality** | **Importance** |
| --- | --- | --- | --- | --- | --- | --- | --- | --- | --- | --- | --- | --- |
| **№ of studies** | **Study design** | **Risk of bias** | **Inconsistency** | **Indirectness** | **Imprecision** | **Other considerations** | **Plate Fixation** | **Intramedullary Nailing** | **Relative (95% CI)** | **Absolute (95% CI)** |  |  |
| Treatment Failure | | | | | | | | | | | | |
| 7 | randomised trials | serious ^1,2,3,4^ | not serious | not serious | not serious | none | 16/298 (5.4%) | 6/327 (1.8%) | **RR 2.19** (0.93 to 5.15) | **22 more per 1000** (from 1 fewer to 76 more) | ⨁⨁⨁◯ MODERATE | CRITICAL |
| All Complications Not Requiring Surgery | | | | | | | | | | | | |
| 9 | randomised trials | serious ^1,2,3,4^ | serious ^5^ | not serious | not serious | none | 106/240 (44.2%) | 52/265 (19.6%) | **RR 2.11** (1.38 to 3.23) | **218 more per 1000** (from 75 more to 438 more) | ⨁⨁◯◯ LOW | CRITICAL |
|  |  |  |  |  |  |  |  | 17.6% |  | **196 more per 1000** (from 67 more to 394 more) |  |  |

**CI:** Confidence interval; **RR:** Risk ratio

1. Lack of blinding across several parameters
2. Lack of adequate randomization and concealment techniques
3. Potential selective outcome reporting
4. High risk quasi-randomized studies included
5. High level of between study heterogeneity

| **OPERATIVE DURATION** | | | | | | | | | | | | |
| --- | --- | --- | --- | --- | --- | --- | --- | --- | --- | --- | --- | --- |
| **Quality assessment** | | | | | | | **№ of patients** | | **Effect** | | **Quality** | **Importance** |
| **№ of studies** | **Study design** | **Risk of bias** | **Inconsistency** | **Indirectness** | **Imprecision** | **Other considerations** | **Plate Fixation** | **Intramedullary Nailing** | **Relative (95% CI)** | **Absolute (95% CI)** |
| Operative Duration | | | | | | | | | | | | |
| 4 | randomised trials | serious ^1,2,3,4^ | serious ^5^ | not serious | not serious | none | 97 | 101 | - | MD **20.64 Minutes higher** (17.34 higher to 23.93 higher) | ⨁⨁◯◯ LOW | NOT IMPORTANT |

**CI:** Confidence interval; **MD:** Mean difference

1. Lack of blinding across several parameters
2. Lack of adequate randomization and concealment techniques
3. Potential selective outcome reporting
4. High risk quasi-randomized studies included
5. High level of between study heterogeneity
